# Workplace Violence Faced by Medical Doctors in Kerala, India

**DOI:** 10.7759/cureus.48887

**Published:** 2023-11-16

**Authors:** Rahul Kunnath, Jayakrishnan Thayyil, Nithin Suresh, Suvarna Soman

**Affiliations:** 1 Department of Community Medicine, Government Medical College, Kozhikode, Kozhikode, IND; 2 Department of Community Medicine, KMCT Medical College, Kozhikode, IND

**Keywords:** kerala, healthcare workers, india, assault against doctors, workplace violence, incidence

## Abstract

Introduction: The escalation of violence against doctors, a global concern, is also evident in India. In recent years, there has been a noticeable increase in the instances of violence against doctors in Kerala, a state situated in the southern part of India. This study examines the prevalence and types of violence against doctors in Kerala, considering factors, such as gender, workplace, designation, timing, and those involved.

Methodology: This cross-sectional study involved modern medicine doctors holding a minimum degree in Bachelor of Medicine and Bachelor of Surgery (MBBS), practicing in Kerala. Data collection used validated questionnaires distributed as Google Forms through WhatsApp and email after obtaining contact details from the Indian Medical Association, Kerala wing. A total of 2,400 doctors across all 14 districts participated, and data analysis was done using the IBM SPSS Statistics for Windows, version 21 (released 2012; IBM Corp., Armonk, New York, United States).

Results: Among 1,948 respondents, 65.6% (n=1279) of doctors experienced violence, predominantly verbal abuse (89.9%, n=1150), and intimidation by gestures (32.7%, n=418). Most incidents happened during the day (84.7%, n=1083), with 32% (n=409) occurring after duty hours. Casualty triage had the highest incidence (57.5%, n=736), followed by outpatient departments (33.6%, n=430). Relatives or bystanders were the foremost perpetrators in 81.5% (n=1043) of cases. Although 48.6% (n=621) of incidents were reported to authorities, only 13.5% (n=173) had any sort of preventive measures taken. A significant 76.7% (n=981) of doctors contemplated relocating abroad.

Conclusion: This research underscores the alarming prevalence of workplace violence against doctors in Kerala, echoing global trends. The inadequate implementation of preventive measures highlights the gap between awareness and action, necessitating an examination of barriers in healthcare settings.

## Introduction

Violence against doctors has been a growing concern in many parts of the world [1-3], including India. Workplace violence (WPV) refers to any act or threat of physical violence, harassment, intimidation, or other disruptive behaviors in a work setting. It can be of various types, including verbal abuse, harassment or threats, physical assaults, and even homicide [4]. WPV can happen at hospitals or outside, such as during home visits for healthcare providers [5]. In recent years, there has been a noticeable increase in the instances of violence against doctors in Kerala, a state situated in the southern part of India [6,7]. In 2022 alone, the state had reported 137 attacks on doctors [8]. Factors contributing to this rise may differ. They include patient dissatisfaction, long waiting times due to inadequate staffing, improper communication, lack of medicines or other adequate facilities, and frustration with the healthcare system [9,10].

Violence against doctors has significant consequences for their physical and mental well-being. It can lead to injuries, trauma, stress, anxiety, and burnout. Such incidents also demoralize doctors, making them question their career choices and affecting their jobs. It also disrupts the smooth functioning of medical facilities. Instances of violence can create an atmosphere of fear and hinder the doctor-patient relationship. It may result in delays in treatment, compromised patient safety, and decreased quality of care. Increasing violence against doctors can make the medical profession less attractive to aspiring doctors. Concerns about personal safety and job security may discourage individuals from pursuing careers in healthcare. Moreover, experienced doctors may leave their positions or relocate to safer environments, leading to a shortage of skilled healthcare professionals [11,12].

Addressing violence against doctors requires a multifaceted approach. It involves implementing preventive measures, such as enhancing security measures at healthcare facilities, improving communication between doctors and patients, promoting awareness about the consequences of violence, and training healthcare professionals on conflict resolution and de-escalation techniques [13-15]. Given these factors, the increasing violence against doctors highlights the urgent need to address this issue comprehensively.

The main objective of our study was to investigate the prevalence of violence experienced by medical doctors at their workplaces in Kerala. We also aimed to examine the various types of violence they encountered and explore factors, such as work timings, place of work, and designations related to the occurrence of such violence. Therefore, the study fills a critical gap in the existing research by explicitly focusing on doctors' experiences in the region.

## Materials and methods

Study setting and participants

This cross-sectional study was conducted with study subjects involving all modern medicine doctors in Kerala who have successfully attained a degree in Bachelor of Medicine and Bachelor of Surgery (MBBS) or above. The comprehensive study period, commencing in July 2021 and concluding in September 2023, entailed ethical committee approval, followed by meticulous data collection, rigorous data analysis, and the exploration of multifaceted factors affecting WPV experienced by doctors. The inclusion criteria involved all modern medicine doctors with a minimum MBBS qualification currently working in all 14 districts of Kerala who were willing to provide data. Doctors not working in Kerala, those unwilling to participate, and doctors in allied medical fields were excluded from the study.

Data collection and sampling method

A convenient sampling method was adopted. To determine the sample size, a study conducted in a tertiary care hospital in Delhi, national capital of India, which reported a WPV of 40.8%, was considered, and this was taken as the anticipated prevalence (P) [16]. An absolute precision (*d*) of 2% was taken, and keeping confidence interval (*Z*) at 95%, the sample size (*n*) was calculated using the formula:

$$n=Z^{2}P(1-P)/d^{2}$$

n = sample size P = anticipated prevalence Z = confidence interval d = absolute precision

Based on this, a sample size of 2400 was established. Subsequently, a semi-structured questionnaire that was validated after pretesting among 30 doctors was introduced to the subjects (n=2400) after making necessary modifications. Questionnaires were distributed to doctors in each of the 14 districts of Kerala in a manner proportional to the number of doctors in each district. This was done through WhatsApp and email after obtaining the district-wise contact details of doctors from the Indian Medical Association (IMA), Kerala register. Various demographic information of the doctors, as well as the details regarding WPV, were collected. 

Data analysis

Data were entered in Google Sheets (Google, USA) and analyzed using the IBM SPSS Statistics for Windows, version 21 (released 2012; IBM Corp., Armonk, New York, United States). A total of 1,948 doctors responded completely to the questionnaire. Data obtained were checked for completeness and consistency and were then coded numerically. Doctors who did not respond or partially responded were excluded from the data analysis. The variables under study included the gender of the doctor, place of work, designation, type and time of violence, and people involved in the violence (their gender, number, and type). We employed a chi-squared test to analyze the association between violence prevalence and both the doctor's gender and their designation. A P-value of less than 0.05 was taken as statistically significant.

Ethical considerations

The research protocol obtained approval from the Institutional Ethics Committee of Government Medical College, Kozhikode (Ref. No. GMCKKD/RP 2021/IEC/141). Each participant was debriefed on the study purpose, and informed consent was taken before participating. The information gathered from the participants was solely used for this study, and utmost confidentiality was maintained to safeguard their identities and data.

## Results

Among the 1,948 responses (81.16% response rate), 45.5% (n=887) were males and 54.2% (n=1057) were females. The majority of respondents were doctors working in government health service departments (31.7%, n=619), followed by doctors working in private hospitals (28.4%, n=554), postgraduate residents (16%, n=312), interns (13.4%, n=261), doctors in medical colleges (8.2%, n=161), and super-specialty residents (2.1%, n=41).

Considering a recall period of 10 years, 65.6% (n=1279) of the doctors had faced violence. Among them, 89.9% (n=1150) experienced verbal abuse, while 32.7% (n=418) faced intimidation by gestures. Threats and blackmailing contributed to 23.8% (n=305), and harassment by police or politicians contributed to 16.2% (n=207). Strikingly, seven doctors had to face sexual abuse, out of which five of them were females (Table 1).

**Table 1:** Various types of violence reported

Type of violence	Frequency	Percentage
Verbal abuse	1150	89.9
Intimidation by gestures	418	32.7
Threats and blackmailing	305	23.8
Harassment by police/politicians	207	16.2
Physical violence	102	8
Cyberbullying	74	5.8
Mob attacks	69	5.4
Sexual attack	7	0.5
Other	8	0.6

Most cases occurred during the daytime (84.7%, n=1083). Still, there had been incidents where doctors were subjected to violence after their duty hours as an aftermath of events during their duty period (32%, n=409).

Regarding specific work settings, doctors in casualty triage had the highest incidence rate at 57.5% (n=736), followed by the outpatient department (OPD) and wards at 33.6% (n=430) and 27.1% (n=347), respectively. Violence in operation theaters and mobile units was less than 1% (Table 2).

**Table 2:** Workplaces where violence occurred

Place of violence	Frequency	Percentage
Casualty	736	57.5
OPD	430	33.6
Ward	347	27.1
ICU	126	9.9
Peripheral centers	112	8.8
Outside workplace	66	5.2
Operation theaters	7	0.5
Mobile units	6	0.5

Information regarding the doctors' designation, gender, and correlation with workplace violence is presented in Table 3.

**Table 3:** Association between violence prevalence with gender and designation of the doctor *Statistically significant

Variable	Faced violence		Total	Chi-square	P-value
	Yes (n=1279)	No (n=669)		5.88	0.053
Gender of the doctor					
Male	607	280	887		
Females	670	387	1057		
Not revealed	2	2	4		
Designation				63.57	< 0.01^*^
Doctors in the health service department	467	152	619		
Interns	140	121	261		
Doctors working in private hospitals	332	222	554		
Postgraduate residents	223	89	312		
Medical college doctors	89	72	161		
Super specialty residents	28	13	41		

As the main perpetrators, 74.8% (n=957) of males dominated the list of instigators. Relatives or bystanders accounted for 81.5% (n=1043) of the individuals involved in violence. Patients were also significantly implicated, comprising 30.3% (n=388) of the cases (Table 4).

**Table 4:** Various perpetrators engaged in violence

People involved in violence	Frequency	Percentage
Relatives	1043	81.5
Patients	388	30.3
Outsiders	360	28.1
Authorities	58	4.5
Co-workers	6	0.5

A near-even distribution existed between single attacks (49.1%, n=628) and mob attacks (50.9%, n=651). Notably, most offenses were committed free-handed (97.4%, n=1246), while a small minority (2.6%, n=33) involved using weapons. Approximately 48.6% (n=621) of the reported cases were brought to the attention of the authorities; however, preventive measures were implemented in only 13.5% (n=173) of these instances. Despite 82.1% (n=1051) of doctors being aware of the legal protections afforded them, only 6.8% (n=87) pursued legal action. Furthermore, 76.7% (n=981) of doctors have contemplated leaving the country.

## Discussion

This cross-sectional study was conducted among medical doctors working in Kerala, a state in the southern part of India. We assessed the prevalence and the type of violence faced by doctors. We also evaluated the relevance of factors, such as the gender of the doctor, place of work, time of violence, designation, and the people involved in the prevalence of violence.

Prevalence of violence 

According to the study, 65.6% (n=1279) of doctors in Kerala have faced violence. Based on research done by Anand et al. in a tertiary care hospital in Delhi (2016), 40.8% of doctors had reported being exposed to violence at their workplace in the last 12 months [16]. A similar trend was noticed in various studies conducted in different parts of the world [1-3]. Our results are consistent with a pan-Indian study conducted by Kaur et al., which suggested an overall percentage of 60-70% in Kerala [17]. The substantial increase in incidents in Kerala indicates a deteriorating doctor-patient relationship, demanding urgent intervention to address the issue.

Types of violence

Similar to other research, verbal abuse remains the predominant form of violence, followed by intimidation by gestures [13,18]. Verbal abuse is often the most common form of violence in healthcare settings due to its ease of perpetration, lower risk of legal consequences, and the power dynamics involved. Patients and their relatives may resort to verbal abuse as an immediate reaction to dissatisfaction, frustration, or fear while interacting with the healthcare system. In addition, stress and high-pressure environments within healthcare facilities can contribute to heightened emotions, further increasing the likelihood of verbal abuse. The high numbers of threats, blackmail, and harassment by police or politicians (23.8%, n=305 and 16.2%, n=207, respectively), along with seven doctors (including five females) facing sexual abuse, can be attributed to factors, such as power imbalances, where individuals in positions of authority or influence exploit their status to exert control or manipulate others, lack of accountability, and underreporting due to concerns about professional repercussions or societal stigma.

Gender 

The distribution of violence is almost evenly split between male (68.4%, n=607) and female (63.3%, n=670 ) doctors, suggesting that both genders are susceptible to violence in their workplace. The lack of a gender difference in violence among doctors may be due to similar occupational risks and equal vulnerability. Similar to other studies [19], a statistical assessment reveals that no significant association exists between the prevalence of violence and the gender of the doctor (P-value=0.053).

Place of work

As reported by prior researchers, a significant portion of the violence was experienced by doctors in the emergency department (57.5%, n=736) [16,20]. There are a few reasons why violence can be more prevalent in these departments. First, casualty departments often deal with many urgent and critical cases. In such high-stress situations, emotions can run high, leading to potential conflicts and aggression toward healthcare professionals. Second, the chaotic nature of emergency departments can contribute to tense situations. Long waiting times, overcrowding, and limited resources can frustrate patients and their families, increasing the likelihood of aggressive behavior. In addition, the unpredictable nature of emergencies means that doctors and staff often face unknown individuals, some of whom may have a history of violence, mental health issues, or substance abuse problems.

This is followed by OPDs (33.6%, n=430), wards (27.1%, n=347), and ICU (9.9%, n=126). Violent events were the least frequent in operation theaters and mobile units (<1%). This decreasing trend in violence across different work settings underscores the need for comprehensive strategies to address the specific challenges posed by each healthcare environment, emphasizing the importance of safety measures and conflict resolution training in high-stress settings, such as emergency departments.

Time of violence 

The violence occurred mainly during daytime hours (84.7%, n=1083), confirming earlier research [21]. Interestingly, 32% (n=409) of these incidents happened after doctors had finished their duty shifts and left their workplace to return home, which dictates that no universal strategy exists to prevent violence. The mere elevation of the security system in the workplace does not necessarily protect healthcare workers from experiencing violence.

Designation

The incidents of violence occurred most frequently among doctors in the health service department (36.5%, n=467), followed by doctors working in private hospitals (25.9%, n=332). Postgraduate residents (17.4%, n=223) and interns (10.9%, n=140) also encountered a substantial share of these incidents. In comparison, medical college doctors (6.9%, n=89) and super-specialty residents (2.1%, n=28) experienced a relatively lower percentage of violence. Notably, the P-value (<0.01) indicates a significant association between the prevalence of violence and the designation of doctors. Nevertheless, it is also plausible that additional factors beyond a doctor's designation contribute to the prevalence of violence. 

People involved in violence

The relatives or bystanders accounted for the majority of individuals involved in violence (81.5%, n=1043); similarly, another study done in Ethiopia showed a comparable trend (55.3%) [21]. This highlights the complex dynamics in healthcare settings, where family members and witnesses may become sources of conflict and aggression. The fact that 74.8% (n=957) of these individuals were males suggests a potential gender-related dimension to the violence. The significant implication of patients themselves in violent incidents (30.3%, n=388) underscores the importance of addressing patient-related factors, such as frustration, fear, or dissatisfaction, in healthcare settings.

Our study encompassed data from doctors with diverse roles in both peripheral and tertiary healthcare facilities, whether in the government or private sectors. The more than 80% response rate from this diverse group of doctors allows us to paint a generalized overview of the prevalence of violence against healthcare professionals in Kerala. The recent approval of the 2023 amendment to the Kerala Healthcare Service Persons and Healthcare Service Institutions Bill by the Kerala State Assembly for preventing violence signals a positive step toward preventing violence against healthcare professionals in the state [22].

The study was primarily centered on doctors when the COVID pandemic was at its peak, proving to be a significant limitation of our study. As a result, the in-person data collection seemed implausible. The data were mainly collected by sending questionnaires through various social media platforms using Google Forms. Considering a recall period of 10 years, memory limitations or inaccuracies may have contributed to underreported information, causing recall bias.

## Conclusions

The research findings highlight the alarming prevalence of workplace violence against doctors in this part of the country, which mirrors global trends. The study underscores the need for urgent attention to this issue and the implementation of comprehensive measures to protect healthcare professionals. Addressing the underlying causes, improving healthcare infrastructure, and enhancing security measures are crucial to creating a safer working environment for doctors. By tackling these issues head-on, the healthcare system can be improved, creating a more conducive environment for healthcare workers to deliver high quality care. The findings of this study will contribute to the existing body of knowledge and serve as a foundation for developing evidence-based policies and interventions to create a safer and more secure working environment for doctors.

Enhancing hospital security involves physical security systems, trained personnel, panic buttons, and collaboration with law enforcement. A strict zero-tolerance policy for violence, offering psychological support to victims, and regularly updating security protocols are also crucial. Introducing and emphasizing interpersonal communication skills during the undergraduate curriculum can positively impact the doctor-patient relationship. Furthermore, future research should explore violence's physical and psychological effects, evaluate existing prevention strategies, and develop interventions to mitigate workplace violence’s impact on healthcare professionals’ well-being.

## Appendices

A Study on Violence Against Doctors in Kerala

This is a study done by Dr. Rahul K, Dr. Jayakrishnan Thayyil, Dr. Nithin Suresh, and Dr. Suvarna Soman.

According to OSHA (Occupational Safety and Health Administration), “Workplace violence is any act or threat of physical violence, harassment, intimidation, or other threatening disruptive behavior that occurs at the work site. It ranges from threats and verbal abuse to physical assaults and even homicide."

By filling this Google form, you consent for participating in this study. Data given here will remain strictly confidential and will be used for this study purpose only. 

Data should be given only by doctors, interns, or residents who are currently working in Kerala and have a minimum qualification of MBBS. Medical students and doctors in allied medical fields such as Siddha, Homeo, Ayurveda, etc. are not included in the study.

Registration number is collected to check the authenticity of the participant and to prevent multiple responses.

This study obtained approval from the Institutional Ethics Committee, Government Medical College, Kozhikode (Ref. No. GMCKKD/RP 2021/IEC/141).

For any further queries, 

E-mail : (corresponding author)

Biodata

1) Registration no. (Kerala State Medical Council):

2) Age:

20-30

30-40

40-50

50-60

Above 60

3) Gender: 

Male

Female

Others

Prefer not to say 

4) Place of work:

Kerala

Outside of Kerala

5) District of work:

Alappuzha

Ernakulam

Idukki

Kannur

Kasargod

Kollam

Kottayam

Kozhikode

Malappuram

Palakkad

Pathanamthitta

Thiruvananthapuram

Thrissur

Wayanad

6) Are you aware of the violences faced by doctors at work?

Yes

No

 7) Have you faced any kind of violence as a doctor in the past 10 years?

Yes

No

**If YES (in question no. 7):**

1) Type of violence (multiple options may be possible):

Physical violence

Verbal abuse

Threats and black mailing 

Harassment by police or politicians

Intimidation by gestures

Cyberbullying

Sexual abuse in any form 

Mob attack

Others

2) Time of violence (multiple options may be possible):

     Daytime: 8 AM to 8 PM shift

     Night time: 8 PM to 8 AM shift

Daytime, at the same time of incident

Daytime, after the incident has happened

Night time at the same time of incident

Night time after the incident has happened

3) Year the incident took place:

2011

2012

2013

2014

2015

2016

2017

2018

2019

2020

2021

Before 2011

4) Designation at the time of incident:

Interns

Post-graduate residents

Super-speciality residents

Medical college doctors

Doctors under health service department

Doctors in private hospitals

5) Did it happen while you were on duty?

Yes

No

6) Place of violence (multiple options may be possible):

Casualty

ICU

OPD

OT

Ward

Peripheral centers

Mobile units

Outside workplace

7) People involved in the offense (multiple options may be possible):

Patient itself

Relatives or bystanders 

Outsiders

Co workers

Authorities 

8) Number of people involved:

Single

Mob attack

9) Gender of people involved:

Female

Male

Others

10) Were weapons used or not?

Yes

No

11) How did you respond to the violence?

Moved legally

Notified to hospital authorities

Out-of-court settlement

Did not respond

Others

12) Were there any preventive measures taken by the hospital?

Yes

No

Don't know 

13) Is there any system available in the hospital to report violence?

Yes 

No

Don’t know

14) Have you ever thought of moving outside India due to increasing violence against doctors?

Yes

No

Maybe

15) Any other suggestions or points to be added:

If NO (in question no. 7):

1) Designation:

Interns

Post-graduate residents

Super-speciality residents

Medical college doctors

Doctors under health service department

Doctors in private hospitals

2) Is there any system available in the hospital to report violence?

Yes 

No

Don’t know

3) Have you thought of taking your service elsewhere due to increasing violence against doctors in India?

Yes

No

Maybe

4) Any other suggestions or points to be added:
